# CCNV: a user-friendly R package enabling large-scale cumulative copy number variation analyses of DNA methylation data

**DOI:** 10.1186/s12859-025-06269-z

**Published:** 2025-09-23

**Authors:** Antonia Gocke, Yannis Schumann, Jelena Navolić, Shweta Godbole, Melanie Schoof, Matthias Dottermusch, Julia E. Neumann

**Affiliations:** 1 Center for Molecular Neurobiology Hamburg (ZMNH), University Medical Center Hamburg-Eppendorf, Hamburg, Germany; 2 Section of Mass Spectrometric Proteomics, University Medical Center Hamburg-Eppendorf, Hamburg, Germany; 3 Institute of Neuropathology, University Medical Center Hamburg-Eppendorf, Hamburg, Germany; 4 IT-Department, Deutsches Elektronen-Synchrotron DESY, Hamburg, Germany; 5 Research Institute Children’s Cancer Center, Hamburg, Germany; 6 Department of Pediatric Hematology and Oncology, University Medical Center, Hamburg-Eppendorf, Hamburg, Germany

**Keywords:** Cumulative CNV, DNA methylation, Copy number variation, Segmentation, EPIC, EPICv2, High-throughput, Chromosomal aberration

## Abstract

**Background:**

Copy number variation (CNV) analyses—often inferred from DNA-methylation data—depict alterations of DNA quantities across chromosomes and have improved tumour diagnostics and classification. For the analyses of larger case series, CNV-features of multiple samples have to be combined to reliably interpret tumour-type characteristics. Established workflows mainly focus on the analyses of singular samples and do not support scalability to high sample numbers. Additionally, only plots showing the frequency of the aberrations have been considered.

**Results:**

We present the Cumulative CNV (CCNV) R package, which combines established segmentation methods and a newly implemented algorithm for thorough and fast CNV analysis at unprecedented accessibility. Our work is the first to supplement well-interpretable CNV frequency plots with their respective intensity plots, as well as showcasing the first application of penalised least-squares regression to DNA methylation data. CCNV exceeded existing tools concerning computing time and displayed high accuracy for all available array types on simulated and real-world data, verified by our newly developed benchmarking method.

**Conclusions:**

CCNV is a user-friendly R package, which enables fast and accurate generation and analyses of cumulative copy number variation plots.

**Supplementary Information:**

The online version contains supplementary material available at 10.1186/s12859-025-06269-z.

## Background

Tumours harbour genetic alterations, some of which include major structural aberrations of the DNA. Such aberrations are associated with changes in DNA quantities across chromosomes, e.g. via deletions or insertions of DNA segments. These changes can be condensed under the term copy number variations (CNVs) [1]. CNVs can be separated into broad aberrations, which encompass whole chromosomes, chromosomal arms or large genomic regions and focal aberrations, which involve only one or few genes and may span only a few megabases. They can additionally be categorised as either gains or losses, depending on whether higher or lower DNA copy numbers are detected.

CNV characteristics can be inferred from DNA-methylation data [2]. Global analysis of DNA-methylation has emerged as a reliable and well accepted method for tumour classification, particularly in the field of brain tumours [3–5]. Recently, the method has also been expanded to many other tumour entities and diseases [6–8]. Moreover, DNA-methylation analyses are already integrated in the WHO classification of brain tumours and are implemented in routine diagnostics [9]. Therefore, this data modality is widely available. A limited number of tools to obtain and analyse CNV data directly from DNA methylation data exist such as the conumee and conumee2 R packages [2, 5].

While whole genome DNA sequencing remains gold standard for CNV analysis, it continues to be an expensive method and is incompatible with formalin-fixed-and-paraffin-embedded (FFPE) tissue [10]. As FFPE represents the standard tissue preservation technique in pathology worldwide, the FFPE compatibility in turn is the greatest advantage of inferring CNVs from DNA methylation data, as this allows access to medical biobanks, especially when investigating rare or heterogeneous diseases [11]. Inferring CNV from RNAseq data has the advantage of being able to analyse multiple levels of information from the same source data (similar to inferring CNVs from DNA methylation data). However, RNA data especially underlies more biases compared to DNA methylation data, as RNA editing, sequencing errors or expression biases can introduce technical and biological noise to the data [12, 13].

With an increasing number of methylation datasets being generated and shared via large databases—according to the FAIR principles—the integration and analysis of larger case series is getting more common [14–16]. This is not only used to validate findings, but also to gain a higher statistical power in the analyses [17]. However, established tools such as conumee are not yet ideal for a large-scale analysis, as the analyses is time-consuming and mainly focussed on singular samples [2].

When analysing CNVs from DNA methylation profiles, the data is reduced by combining several DNA-Methylation sites to so called segments based on similar quantification of DNA material [18]. A current limitation when analysing multiple samples with existing tools is, that they do not result in the same segment constraints. Instead, segment sizes and limits are set differently in each sample based on individual data characteristics [2, 18, 19]. This approach captures sample specific differences but interferes with downstream analysis and the efficient generation of cumulative CNV plots.

Another challenge of CNV analyses is the accurate identification of focal DNA aberrations. Such aberrations of single genes may have a high clinical relevance for the diagnosis of different tumour entities, for prognostic stratifications (e.g. CDKN2A/B), or for the identification of therapeutic targets [3, 20–24]. As the small size of such aberrations increases the risk of being overlooked or disappearing in noise, focal aberrations are especially difficult to analyse when investigating multiple samples simultaneously [25]. Due to the high impact of focal DNA alterations on classification, the course of the disease and the treatment, a correct identification is essential and available CNV tools should be evaluated concerning accuracy and sensitivity when calling such aberrations. Easily accessible test sets, however, do not yet exist.

To address the aforementioned challenges, we established the CCNV tool, an easy-to-use R package, which allows for thorough and fast CNV analysis of multiple samples simultaneously. The tool does not only provide established cumulative plots, which show the frequency of certain aberrations, but also introduces a novel type of plot, displaying the intensity of the aberrations across the investigated samples.

We combine enhanced noise reduction with different segmentation algorithms and an optional sorted data-frame output enabling easy downstream analysis. To allow for a fast and accurate CNV analysis on all common DNA-methylation arrays and ensure broad user acceptance we integrated the established CNV packages conumee and conumee2 in our package and users can switch between two major modes depending on their needs.

The first mode is the sample-wise (SW) approach, where each sample is segmented consecutively with established methods, and visualised with the GenVisR and ggplot2 packages [2, 18, 26, 27]. This approach retains the sample heterogeneity and visualises all samples accurately and provides an easy one-function-method to generate cumulative CNV plots.

The second mode is the combined segmentation (CS) approach and allows to simultaneously segment multiple samples using an enhanced penalised least squares regression algorithm, that has not yet been applied to methylome-inferred CNV analysis before [28]. The results are also visualised using the ggplot2 package [27]. CS enforces segment homogeneity throughout all samples and achieves a significant speed-up in the analysis.

Further, the package encompasses an optional function for returning a data frame which gives an overview of all found chromosomal characteristics in all samples ordered based on their similarity which allows easy downstream analysis. We further propose a Monte Carlo simulation-based approach, to rigorously validate CNV tools concerning the identification of different focal aberrations. CCNV is thus thoroughly validated on simulated and real-world data. Finally, runtime measurements revealed a significantly reduced computing time applying the CS mode.

Taken together we present an accurate, flexible, fast and easy-to-use tool for CNV analyses from DNA-methylation data enabling an efficient analyses of large data case series.

## Implementation

### Overview

**Fig. 1:**
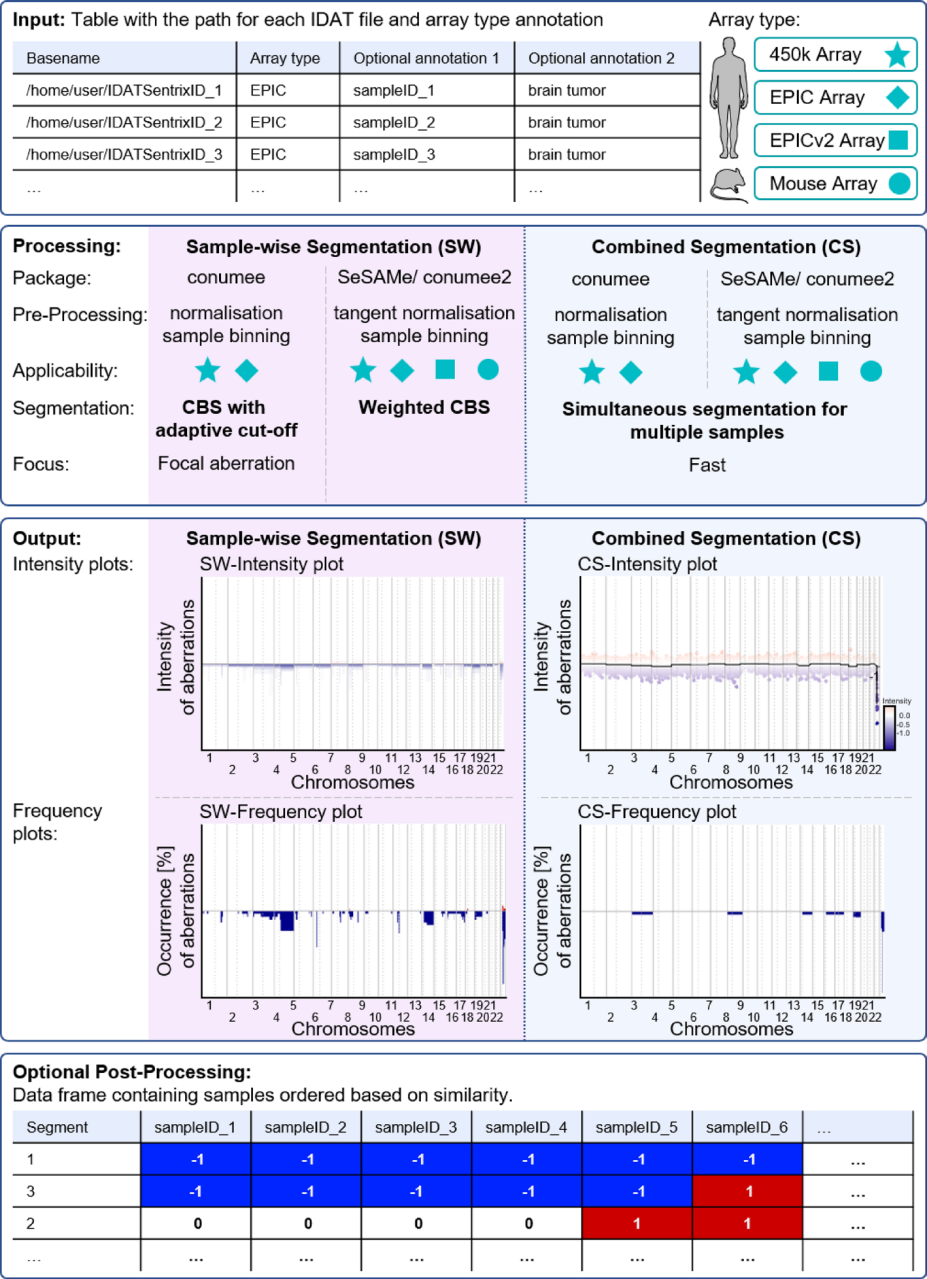
Overview of the workflow of the R package, divided into “input”,” processing”, “output” and “optional post-processing”. The structure of the input file which can refer to all common DNA-methylation arrays is depicted. For processing the user can decide between the modes “sample-wise copy number segmentation” (SW) or “combined copy number segmentation” (CS) focusing either on single samples (SW) or efficient handling of multiple samples (CS). The output shows exemplary plots visualized in each mode. The optional post-processing gives a similarity-sorted dataframe with respective segments and features “1 = gain”, “0 = no alteration” and “− 1 = loss”. An exemplary visualization with gains (red colour) and losses (blue colour) is added to the table. CBS = circular binary segmentation

CNV analyses can be derived from DNA-methylation data based on the principle that copy numbers can be inferred from the summed intensities of the measured methylated and unmethylated DNA loci [2, 18]. Our main workflow is separated into three main steps, 1) reading the data (input), 2) data processing and 3) providing the output as CNV plots (Fig. 1). An optional fourth post-processing step generates a comprehensive output as a data frame providing all samples reordered based on their similarity concerning CNV alterations. This step can be added via the *get.chromAberrations* – function implemented in the package.

The input data is a table containing information about the path to the DNA methylation data raw files, the so called.idat-files and the respective array type. After reading the table, the *cumul.CNV*—function automatically reads in the array type for the case series and determines, whether conumee or conumee2 needs to be used. If the input data is compatible with both versions of conumee, the former will be used as a default setting. After reading the.idats, the next step is data processing. If multiple array types are combined into one large cohort, they are additionally processed using mLiftOver, integrated in openSesame [29].

During processing, the files are read, normalised against control samples and the CpG-sites are binned into larger genomic parts using the appropriate version of conumee, before being segmented. By default, both segmentation modes (sample wise (SW) and combined segmentation (CS)) are used. However, the user can specify whether only one or the other segmentation mode should be used.

The standard outputs are CNV plots, differing with respect to the segmentation mode (SW or CS). Each mode has one corresponding intensity plot, displaying the strength of the aberrations, and one corresponding frequency plot, displaying how often aberrations occur across the case series (for details see “Visualisation”). The intensity plots are a new development, which can be read intuitively, like classical CNV plots. The frequency plots rely on identifying aberrations based on set thresholds and only these are used to compile the plots. Additionally, the user can specify, whether the segmentation data should be returned as a data matrix containing the intensity for each segment and for each sample.

In the SW mode segments are defined for each sample individually and thus, identical segments cannot be identified. Therefore, the optional post-processing step returns a data matrix that condenses the CNVs to the chromosomal arms, by computing the weighted mean. Aberrations are defined based on a settable threshold, either as a gain (1) or as a loss (− 1), or as no change (0). In the CS approach, the standard is to identify the aberrations for each determined segment, instead for the chromosomal arms. The function *chrom.arm* = *TRUE* however, allows to calculate the aberrations based on the level of chromosomal arms.

### Data Preparation

Our tool can use DNA methylation data generated from Illumina’s Infinium arrays: HumanMethylation450 (450 k, > 450,000 probes), MethylationEPIC (EPIC, > 850,000 probes), MethylationEPIC v2.0 (EPICv2, > 935,000 probes) and Mouse Methylation (mouse, > 285,000 probes). Raw IDAT file names can be provided as a table via their file path and with information of the corresponding array type. It will then be directly imported using the minfi package [30] for 450 k and EPIC arrays - or else using the sesame package [31] for mouse arrays, EPICv2 arrays and case series with a combination of different arrays. If different array types are combined, genomic liftover is performed automatically using mLiftOver [29]. The case series is always projected onto the lowest array type used. Probe annotations are loaded from the conumee [2] and conumee2 [18] packages.

#### Data normalisation

Our tool provides two different types of normalisation depending on the conumee version used. Each technique normalises the query samples against a set of control samples (see below). In conumee, the probe intensities are normalised by fitting a linear model to the raw signal intensities and a calculated log2-ratio is then used further [2]. In conumee2 a tangent normalisation is applied to the signal intensities. Here the log2-ratio of the probe intensities of the query sample versus the linear combination of control samples is calculated and used for further analysis [18]. If 450 k or EPIC arrays are analysed – also in combination with other array types- publicly available reference samples from the minfiData and minfiDataEPIC packages are used, however reference samples can also be supplied to the package via a description table. For analysing only EPICv2 and mouse arrays there are no publicly available control samples and they need to be supplied to the package.

#### Genomic binning

After normalisation, genomic binning is performed using the default functions of the conumee [2] or conumee2 [18] package to reduce the complexity of the data. Here the algorithm splits the genome into parts of a defined size (50 kb) and iteratively merges the parts until bins with a minimum number of 15 probes are assembled. The median log2-ratio of the probes within that bin is then assigned as the intensity of that bin.

#### Segmentation

Three different segmentation algorithms are employed in the CCNV package. The circular binary segmentation (CBS) and the weighted CBS, implemented in the conumee [2] and conumee2 [18] package, respectively, are used to consecutively segment each sample. The penalised least squares regression algorithm, implemented after Nilsen et al. [28], segments all provided samples simultaneously (CS mode).

Conumee [2] utilises a circular binary segmentation [32] to segment bins into regions of the same copy number quantity. The CBS treats the genome as a circle and recursively tries to partition the genomes into different intervals maximising the difference in partial means. Once a significant difference is identified, the algorithm is applied to the newly defined intervals.

Conumee2 [18] applies a modified CBS version, the weighted CBS [32], to reduce the noise in the data. Here, a weight inverse to the variance of the normalised probe intensities within the bins is assigned to each bin, reducing the impact of bins with a high variance.

The penalised least squares regression is implemented after the fast “Piecewise Constant Fit”—algorithm for multiple samples as proposed by Nilsen et al.[28]. In our implementation, the genome is separated into the different chromosomes, to circumvent the identification of CNVs spanning across chromosomes, and the algorithm is applied to each chromosome individually. Two high pass filters are applied to heuristically determine potential breakpoints and the cost, including the breakpoint penalty gamma for each segment is calculated and stored. The cost is then minimised to determine the breakpoints and segment averages (the intensities of the CNV) are determined. Larger penalties (representing a higher gamma – value) lead to less sensitive detection of segments. Considered gamma values can be set by the user.

### Visualisation

The cumulative copy number variation plot (CCNV) or CS-Intensity plot displays the mean intensities per bin as a jitter plot, overlaid with the mean values of each segment. It does not reflect how often an aberration occurs, only the intensity (copy number). The CS-Intensity plot can be combined with gene annotations. Here a list of gene names needs to be provided. The probe annotations are used to match the gene names with the bins corresponding to their gene regions. The bin with the highest number of probes representing the gene region is used to mark the gene. The gene is only annotated if its bin has an intensity value of ≥ 0.15 or ≤ − 0.15.

The SW-Intensity plot reflects the calculated intensities per segment for each sample. The segments are displayed as rectangles with an opacity dependent on the number of samples. The more a segment or changes of the segment occurs, the more visible it will be.

1 $${opacity}_{seg}=1\div number \, of \, samples$$

The CS-Frequency plot depicts how often an aberration occurs across the investigated case series. An aberration is identified, if the mean value per segment exceeds the fixed threshold θ (default 0.2). To further reduce false positive calling of aberrations due to noise, the threshold is additionally multiplied by the calculated noise per segment for samples being segmented with the CBS algorithm (Suppl. Figure 4). Additionally, segments are filtered to contain at least two bins per segment.

2 $${\theta }_{CBS}=\theta \times {noise}_{seg}$$

The SW-Frequency plot is generated with the help of the GenVisR package [26], which generates new smaller segments from overlapping segments identified in the segmentation algorithm. The visualisation of the plots was done using the ggplot2 package [27].

### Validation and Benchmarking

#### Accuracy

To validate whether the CS can capture focal aberrations as successfully as the SW segmentation, all possible visual outputs were generated (SW + conumee (SW_Cv1), SW + conumee2 (SW_Cv2), CS + conumee (CS_Cv1), CS + conumee2 (CS _Cv2)) and compared for methylation data of the brain tumour entity ATRT-MYC from the data case series published by Capper [5].

To compare, whether the combined segmentation generates similar results to the single sample segmentations, the mean intensity for each chromosome was calculated. If multiple segments occurred, the weighted mean based on the segment length was calculated. Both segmentations were then compared by calculating the Pearson Correlation Coefficient for each sample.

To verify if the segments calculated by the combined and sample-wise segmentation yielded sensible results, the tool was tested on the methylation data published by Capper et al. [5] and on oligodendrogliomas with known broad copy number aberrations [33]. Lastly to verify the algorithm on multiple array types, the algorithm was also tested on pituitary tumours [34] and a newly generated mouse model [35].

To analyse CNV accuracy concerning focal aberrations in a standardised manner, we conducted Monte Carlo simulations [36] at the HSUper computing cluster (571 compute nodes each equipped with 256 GB RAM and 2 Intel Icelake sockets; each socket features a Intel(R) Xeon (R) Platinum 8360Y processor with (up to) 36 cores, yielding a total of 72 cores per node) (1000 iterations, minimising SLURM scheduling failures to < 5%). We investigated how well the CS segmentation captures different types of focal amplifications, dependant on the gamma-value. For each simulation all control samples published by Capper et al. [5] were used as a basis and we randomly sampled a gene (from all genes captured on the 450 k array), a frequency of the aberration (from [0.2, 0.4, 0.6, 0.8 1]) and a strength of the aberration (from [+ 0.5, + 1, + 1.5, + 2]). After determining the respective bins for the genes, we added + 3 bins on each side of the region, increasing the number of bins per aberration to a minimum of 7, to simulate “real life” aberrations, as known focal SMARCB1 losses or focal MYCN amplifications usually encompass 7–11 bins, and increased the intensities of these bins in the determined proportion of the data by the determined strength of the aberration.

We then plotted the observed aberration of samples, against the expected and determined an aberration as found, if the segment of the expected bin length was identified by the segmentation algorithm, even if the expected frequency could not be observed. Lastly, we also investigated how often aberrations were found or missed based on their strength and frequency.

#### Runtime

To benchmark the different algorithms, runtime measurements were performed on the publicly available data from Capper et al. [5] accessed via NCBIs Gene Expression Omnibus with the accession number GSE109381. The data was separated into the published tumour subtypes (n = 92) and normalised against the publicly available data from the minfi package [30]. Each data point for the runtime measurements was acquired using an exclusive reservation of a regular compute node if the supercomputer HSUper, each equipped with 256 GB DDR4 RAM and 2 Intel(R) Platinum 8360Y. Computations were submitted as an array with up to 30 parallel jobs and all data was read from a BeeGFS file system with a read-bandwidth of approx. 1 GB/s.

## Results

### Validation and benchmarking

**Fig. 2:**
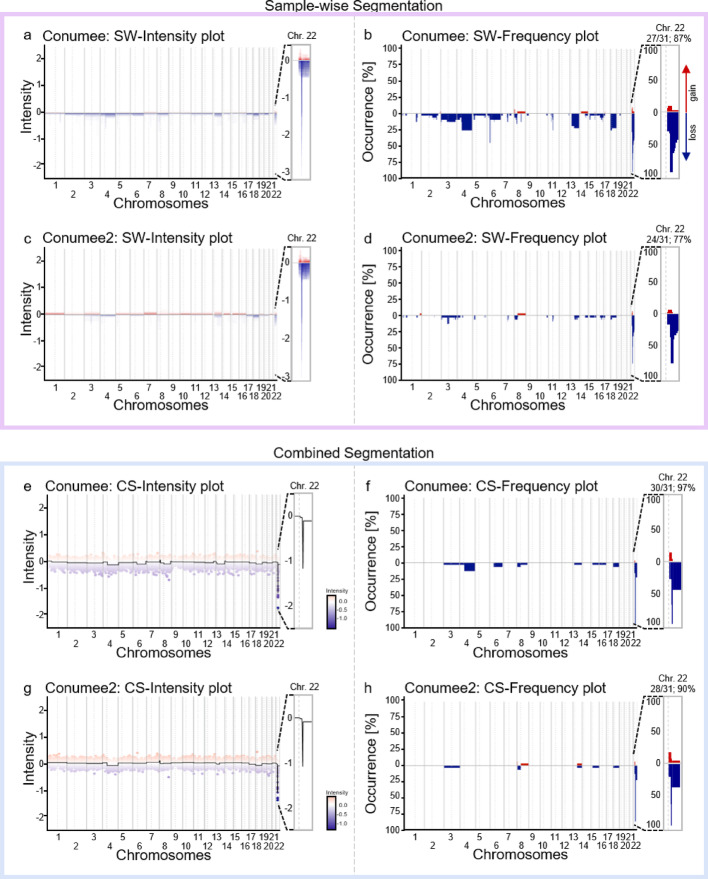
Possible outputs shown for the brain tumour entity ATRT-MYC (n = 21), for SW mode (**a**–**d**) and CS mode (**e**–**h**), either processed with conumee (**a**, **b**, **e**, **f**) or conumee2 (**c**, **d**, **g**, **h**). The expected focal loss of the SMARCB1 gene on chromosome 22 was found best in CS mode and conumee (**b**, 97%). Respective chromosomal region on 22q is shown as a zoom in for all panels

The embryonal brain tumour entity ATRT (atypical teratoid rhabdoid tumour) is an aggressive embryonal brain tumour and can be classified into three different subgroups based on molecular characteristics. The ATRT-MYC subgroup typically harbours a SMARCB1 loss on chromosome 22q [23]. For the validation of our tool, we applied both the CS and SW segmentation method on publicly available ATRT-MYC data (n = 31, [5]), and show that the expected SMARCB1 loss can be detected reliably as a focal loss with both segmentation algorithms. As ground truth, CNV plots from single sample analyses were manually inspected and interpreted by two board certified neuropathologists. The loss of SMARCB1 was identified more often using the novel CS mode compared to the SW mode and when using the established algorithm integrated in R package conumee compared to R package conumee2 (CS-conumee: 30/31; 97% | CS-conumee2 28/31; 90% | SW-conumee: 27/31; 87% | SW-conumee2 24/31; 77%, Fig. 2).

**Fig. 3:**
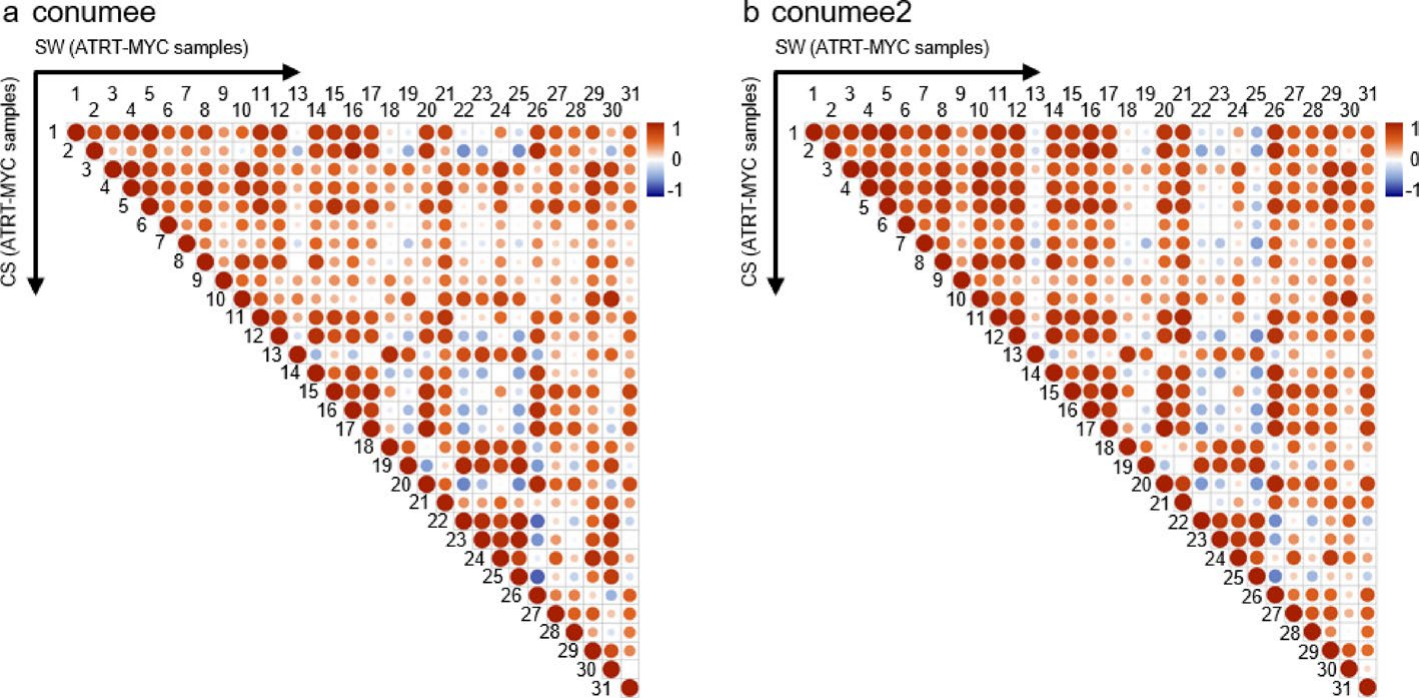
Correlation analysis between combined (CS) and sample-wise segmentation (SW). Calculated sample wise pearson correlation between the 31 ATRT-MYC samples published Capper et al. (2018) segmented using the combined segmentation with the PCF algorithm and the sample-wise segmentation using CBS implemented in conumee (**a**) and implemented in conumee2 (**b**)

To directly compare CS and SW mode, the correlation between the chromosomal weighted mean intensities of all investigated ATRT-MYC samples (n = 31, [5]) was calculated either in CS or SW segmentation mode. The analysis reveals, that CS and SW mode showed very high sample wise correlation between the segmentation results, regardless their pre-processing methods of conumee (mean Pearson correlation = 0.995, Fig. 3a) and conumee2 (mean Pearson correlation = 0.993, Fig. 3b).

**Fig. 4:**
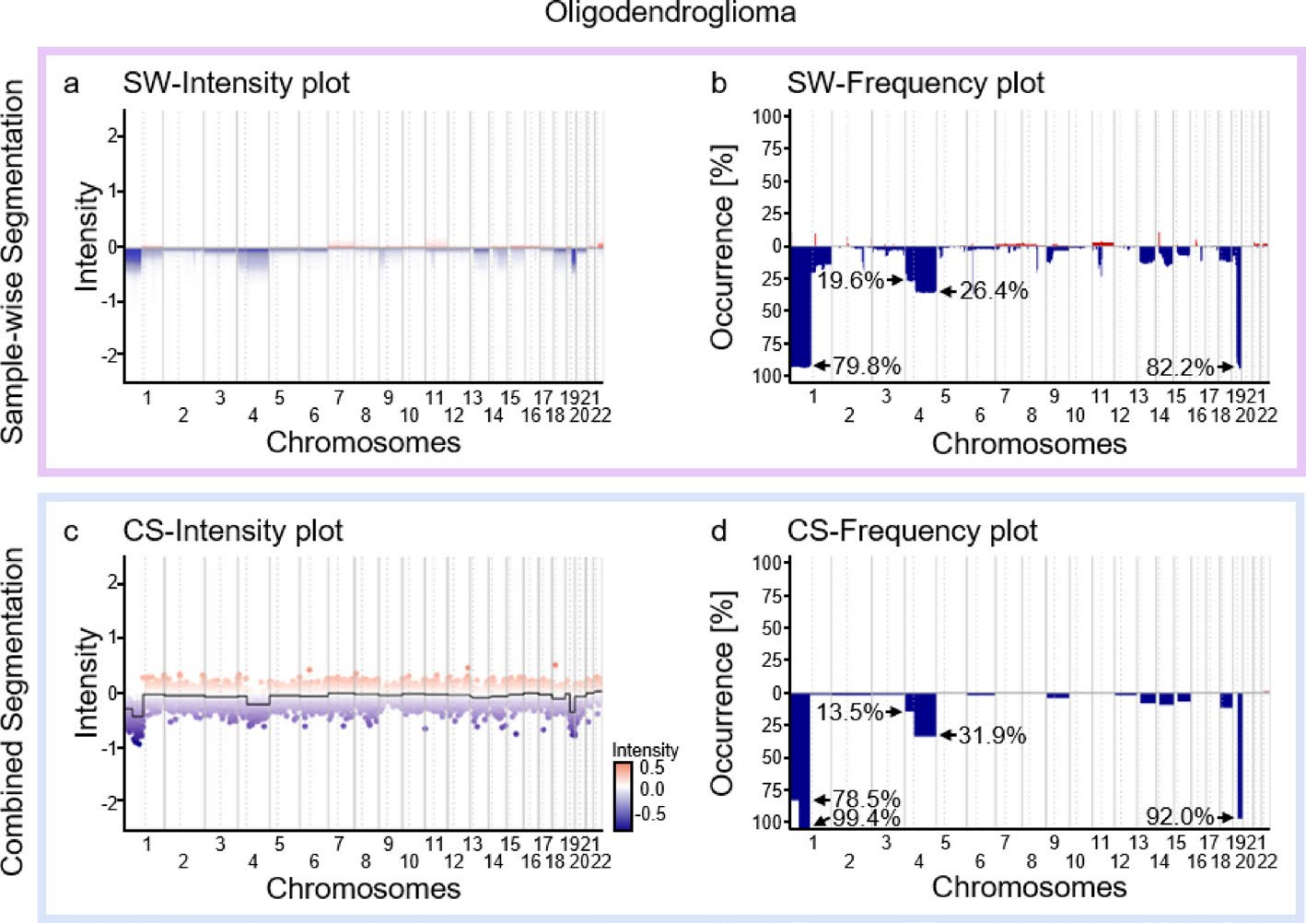
Intensity (**a**, **c**) and frequency (**b**, **d**) plots for the CS (**a**, **b**) and SW (**c**, **d**) segmentation modes for a dataset of oligodendroglioma (n = 163, (5)). Conumee version 1 was used for the preprocessing

Further a dataset for oligodendroglioma was used (n = 163, [5]), a tumour entity defined by a characteristic whole arm loss of the 1p and 19q chromosomal arms [37]. Of n = 163 analysed tumours 1p/19q loss was detected most using CS compared to SW (CS-1p.1 (left segment): 128/163; 79% | CS-1p.2 (right segment): 162/163; 99% | SW-1p: 130/163; 80% || CS-19q: 150/163; 92% | SW-19q: 134/163; 82%, Fig. 4a–d), whereas the loss of chromosomal arms 4p/4q was observed less often (CS-4p: 22/163; 14% | SW-4p: 32/163; 20% || CS-4q: 52/163; 32% | SW-4q: 43/163; 26%, Fig. 4a–d). The frequency of the aberrations were determined using the *get.chromAberrations*—function and a threshold of 0.2. Further testing was conducted on different types of brain tumour data sets (n = 3905 representing 92 distinct brain tumour entities (see data availability section and [5]) and two more available array types (mouse array, Suppl. Figure 1, EPIC (34), Suppl. Figure 2) from publicly available data (34, 35, 38–40). For the EPIC Arrays, we could show that our R package can emulate the trends of the published cumulative plots, differences that occur regarding the intensity of the CNVs may be attributed to different control samples used.

**Fig. 5:**
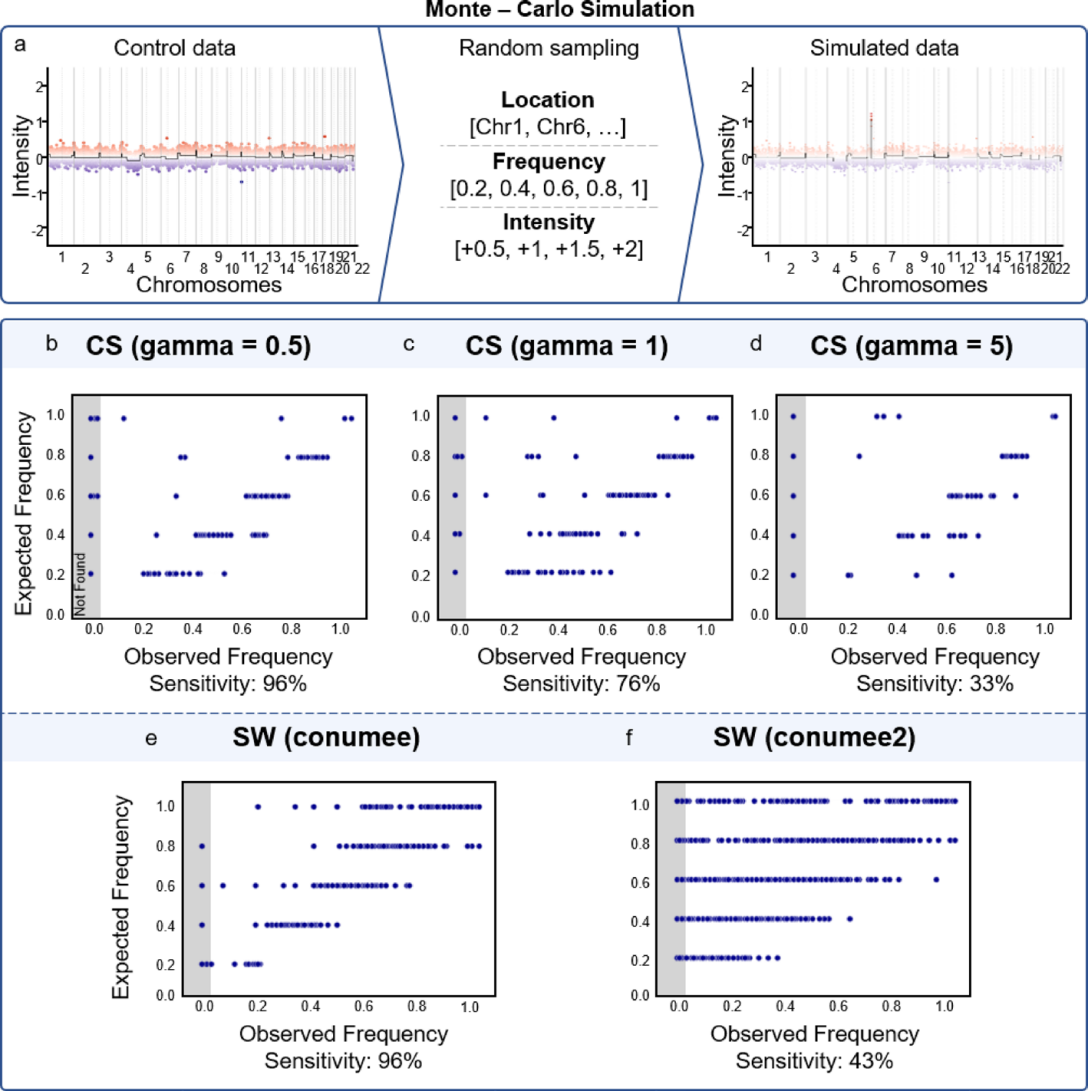
Overview of the Monte Carlo Simulation approach for simulating focal aberrations (**a**) and validation results for CS mode, tested for three different gamma values (**b**–**d**) and SW mode for conumee and conumee2 preprocessing (**e**, **f**). Observed versus expected frequency plotted in approximately n = 1000 simulations including different frequencies and intensities

The above shown examples show limitations of “ground truth” data as there is debate regarding appropriate cut-offs for relevant gains or losses [41, 42]. Samples may show lower intensity deviations, such that these may not be regarded as losses (or gains) based on a given cut-off. Thus, to validate the ability of the CS algorithm to identify focal aberrations from DNA methylation data in a controlled manner and with a clear set ground truth, we tested its applicability on a simulated dataset. Using a Monte Carlo approach, we simulated the chromosomal location, strength and frequency of an aberration in control sample data (reflecting basic noise, n = 119, [5]) and validated the accuracy of the CS algorithm in relation to different gamma—values. In the CS mode, the gamma—value can be defined by the user and represents the breakpoint penalty. Lower gamma values lead to a higher sensitivity, which was confirmed by our simulations, as aberrations were identified more often for low gamma values (Fig. 5) [28]. Additionally, the more pronounced an aberration is and the more often it occurs in the dataset, the more likely it was to be found. With gamma = 5 only strong aberrations with an intensity change of ≥ 1.5 or if they occur in > 60% of the samples were identified reliably. For lower gamma values (≤ 1) weaker and less often occurring aberrations were also be identified reliably (75% correct identification for gamma = 1 and 98% correct identification for gamma = 0.5, Suppl.Fig. 3). For the newest EPICv2 arrays, the highest sensitivity can only be achieved with the CS algorithm (98%), as the SW algorithm employing the conumee2 processing only shows a sensitivity of ca. 43%.

No false positives were identified for a gamma value of 5, and with gamma = 1 one loss on chromosome 22 was identified, which was however filtered automatically by the package using the minimum aberration width of two bins. For gamma = 0.5, six additional aberrations were identified in 986 of all 988 simulated cases (7 and 8 aberrations in the remaining two cases, respectively). Again, after the package’s default filtering for at least two bins per aberration, two aberrations were eliminated from further analysis in each simulation. The remaining aberrations exceeded the cut-off in max. 50% of the samples (max. 27% for increased threshold of 0.3 – showing a false positive aberration that results from the noisy control data).

In conumee2, up to 25 additional aberrations were identified, and none were eliminated after filtering for at least containing two bins. However, each aberration only occurs in up to seven samples (6%). In conumee, 46 to 59 aberrations were found additionally in the simulations, and they occurred in up to 12% of the samples.

**Fig. 6:**
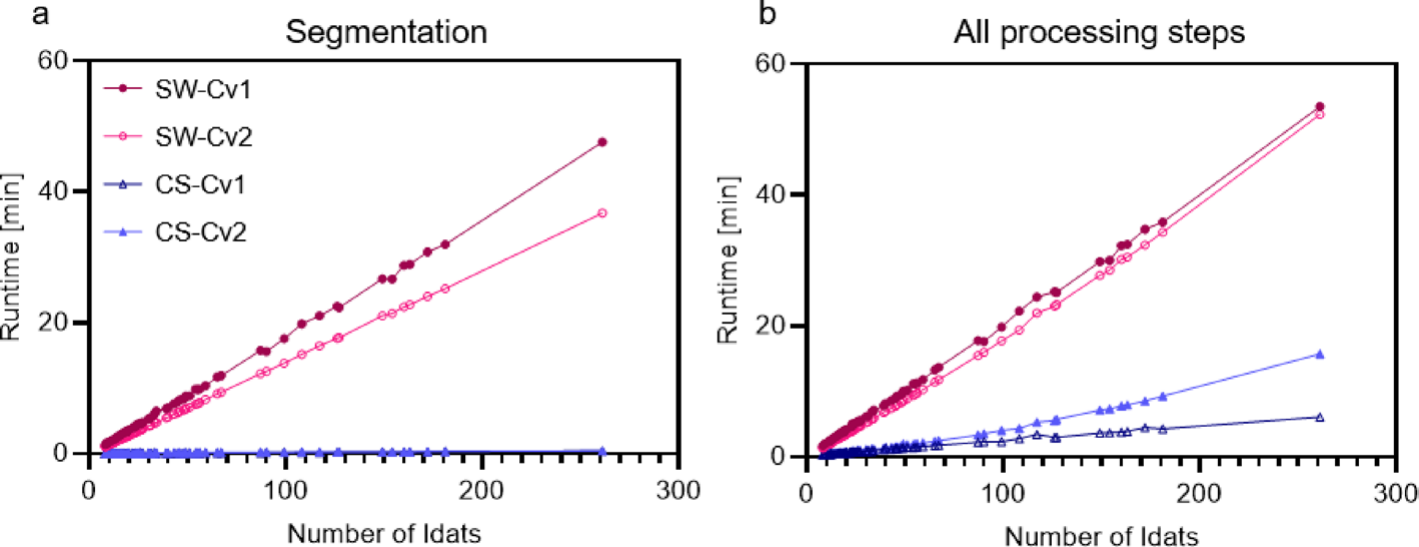
Runtime measurements of the different combinations of segmentation algorithms. SW mode using conumee (SW-Cv1) or conumee2 (SW-Cv2) or CS mode using conumee (CS-Cv1) or conumee2 (CS-Cv2) for the preprocessing on the different brain tumour subtypes published by Capper et al. (2018) (n = 92 entities, n = 3905 samples). The runtime was measured for just the segmentation algorithm (**a**) and for all processing steps (**b**)

As analysing large datasets requires fast and efficient algorithms, we next investigated the runtime of the CS mode within the CCNV package in comparison to SW mode. Using the (standard) SW segmentation algorithm, runtime increased linearly with increasing sample number (up to n = 258) [5]. In contrast, the runtime of the CS algorithm showed a near constant behaviour, when increasing the number of samples (Fig. 6a). As all samples are normalised and binned using conumee or conumee2, the runtime of the complete function for the CS algorithm also increases linearly with the number of samples, however at a much slower rate compared to the complete function using the SW algorithms (Fig. 6b).

To sum up CCNV represents a major advancement of CNV analysis providing accessible and efficient tooling with high sensitivity.

Our software tool provides significant improvements of usability and accessibility of CNV analyses (e.g., min. 4 packages and 13 lines of code using standard tooling for a frequency plot vs 1 line of code with our package, see GitHub for code examples) and allows to seamlessly combine different versions of methylation arrays (Table 1).

**Table 1:** Overview of functionality of CCNV packages compared to existing packages

Category	CCNV: CS	CCNV: SW	Existing packages:	
			conumee	conumee2
Visualisation	Intensity Plot, resembling classical CNV plot and Frequency Plot	Intensity Plot, resembling classical CNV plot and Frequency Plot	In combination with GenVisR package: Frequency Plot	Frequency Plot
Usability	1 package 1 line of code	1 package 1 line of code	Minimum 5 packages	Minimum 4 packages 13 lines of code
Segmentation	Implementation of enhanced PLS segmentation algorithm for CS approach	(Weighted) circular binary segmentation (SW)	Circular binary segmentation (SW)	Weighted circular binary segmentation (SW)
Runtime	CS: 200 idats: 5 min	SW: 200 idats 40 min	SW: 200 idats 40 min	SW: 200 idats 40 min
Sensitivity (for focal aberrations)	Up to 96% sensitivity for all array types	Up to 96% sensitivity (for 450 k and EPIC arrays) Up to 43% sensitivity (for EPICv2 arrays)	Up to 96% sensitivity (for 450 k and EPIC arrays)	Up to 43% sensitivity (for EPICv2 arrays)

It represents the first package to date that provides users with cumulative intensity plots, more similar to the classic CNV plots of single samples, which renders it of particular interest in research and diagnostics. While CCNV provides users with a more accessible interface to the current (sample-wise) gold-standard tools, we implemented an enhanced algorithm for combined segmentation that greatly improves runtime and scalability (e.g. 8 times faster for 200 idats, Fig. 6) while preserving analysis results for suitable hyperparameter values, which we showed in various validation sets (comparable sensitivity, Fig. 5, Table 1).

## Discussion

Here we present CCNV, an R package that is able to generate cumulative copy number variation plots (CCNVs) inferred from DNA methylation data. It provides flexible, comprehensive, fast and user-friendly functions for clinicians, wet-lab researchers and bioinformaticians alike, to easily characterise and understand large sample case series based on their copy number variation profiles. This can be achieved by automatically reading and normalising data directly from the raw .idat files and generates two plots, one displaying the intensities and one displaying the frequency of the aberrations. Additionally, it also includes an optional function, that reorders all samples within a case series together based on their similarity and returns a comprehensive overview of aberrations.

Previously published packages could already generate cumulative plots and show the frequency of the aberrations in sample case series [18, 19, 26]. However, the consecutive segmentations of the data were time consuming and the user had to use a cumbersome combination of different packages or web tools [18, 19, 26, 30]. Generating a cumulative CNV, thus required a higher level of bioinformatic expertise and the option to generate plots displaying the intensity of the CNVs was limited. Additionally, the heterogeneity of the samples resulted in inconsistent segments, complicating a comparable overview of similar aberrations across the complete sample case series. In contrast, the benefit of investigating every sample individually is that it ensures the detection of aberrations occurring in only a small number of samples, despite the fact that this approach is time-consuming.

With the newly implemented algorithm, multiple samples can be segmented simultaneously, leading to consistent aberration restrictions across the whole case series. The considered homogeneity within the samples highlights the areas of the genome which are consistently altered across the whole case series. Additionally, the implemented intensity plot intuitively visualizes aberration frequencies and allows for the identification of well-defined aberrations. Data simulations with controlled focal aberrations showed that even aberrations occurring in the minority of samples can be reliably identified with suitable parameters. However, lower gamma values—resulting in increased sensitivity- come to the cost of decreased specificity and one has to choose carefully, taken the sample quality and data noise into account (Suppl. Figure 4). The trade-off between sensitivity and false positives can be observed in both the CS and the SW algorithm, as conumee also shows greater sensitivity than conumee2 regarding the identification of focal aberrations, but displays much higher false positive rates. Another important influence on the detection of aberrations is the cut-off. However, since there is no universally agreed on cut-off, the selection of such a cut-off remains the responsibility of the user [41, 42]. This difficulty is aggravated by the fact, that the intensity is also influenced by the tumour type and the purity of the tumour samples, as well as the control samples against which the analysed samples are normalised. This difficulty in choosing the correct cut-off can be investigated in the oligodendroglioma samples (Fig. 4), as one sample did not exceed the cut-off of − 0.2, as the intensity was detected as − 0.193. Nevertheless, this sample was identified as oligodendroglioma by both manual and molecular analysis.

Of note, due to the segments having the same aberration restrictions, the frequency loss of SMARCB1 in the ATRT-MYC data set could be better represented in the homogenised frequency plot. In the individualised frequency plot, lost segments in SMARCB1 were often not allocated to the exact same region and thus not regarded as a similar alteration.

Focal aberrations are known to be of a high clinical importance and to have a large impact on the diagnosis and treatment of cancers [1, 21, 43]. We propose a new method of rigorously benchmarking focal aberrations for CNV tools using a Monte Carlo Simulation and testing for different chromosomal locations, frequencies and amplitudes of respective aberrations. This can help to validate the algorithm’s sensitivity and specificity when identifying and characterising focal aberrations.

Although substantial run-time improvements could already be facilitated through the implementation of the CS algorithm, the potential for further enhancements for both the CS and SW algorithm persists. Potential achievements could be realised through the parallelisation of different processing or pre-processing steps, which will be further explored in subsequent studies.

Finally, our additional facultative post-processing function allows to investigate the heterogeneity across samples in a quick way and to identify possible subclusters with shared CNV features in a large, analysed case series. This will enable a fast stratification of patients in future clinical and molecular datasets.

## Conclusions

The generated R package provides flexible, comprehensive, fast and user-friendly functions for a wide range of users to easily characterise and understand large sample case series based on their copy number variation profiles (Table 1).

The package integrates the established conumee algorithms, can work with all common DNA methylation array types and provides two main options: 1) SW mode displays each aberration sample specifically and thus gives a complete but heterogenous overview of the whole case series, whereas 2) CS mode quickly and efficiently identifies specific areas that are consistently changed across the whole case series, leading to enhanced CNV calling and reduced noise, whilst displaying a high sensitivity (Table 1). Additionally, the CCNV package also generates cumulative CNV plots displaying the intensity and the frequency of the aberrations across the investigated samples, providing a deeper insight into the data (Table 1). Thus, the new CCNV R package enables comprehensive, fast and easy-to-use cumulative CNV analysis of large, complex datasets, generating concise and paper-ready graphs for a multitude of users.

## Availability and requirements

Project name: CCNV (Cumulative Copy Number Variation). Project home page: https://github.com/Neumann-Neurooncology/CCNV. Operating system(s): Platform independent. Programming language: R. Other requirements: R 4.4 or higher. License: GPL-2.0 open-source license. Any restrictions to use by non-academics: no restrictions.

## Supplementary Information

Below is the link to the electronic supplementary material.

Supplementary Material 1.

## Data Availability

Previously published methylome data was included from GSE109381 [5], GSE246645 [27], GSE240627 [28], E-MTAB-7762 [30], GSE226764 [31] and from GSE207937 [29]. The segmentation data of all cases from GSE109381 [5] separated by their subtype can be accessed via the validation branch on GitHub (https://github.com/Neumann-Neurooncology/CCNV).

## List of abbreviations

CS: Combined Segmentation
SW: Sample – Wise segmentation
CCNV: Cumulative Copy Number Variation
