# Supplementary material for: CCNV: a user-friendly R package enabling large-scale cumulative copy number variation analyses of DNA methylation data
Source: BMC Bioinformatics. 2025 Sep 23;26:229. doi: 10.1186/s12859-025-06269-z (PMC12459049; doi:10.1186/s12859-025-06269-z)
Supplement: Supplementary file 1 — Supplementary Material 1. [file 12859_2025_6269_MOESM1_ESM.pptx]

## Slide 1
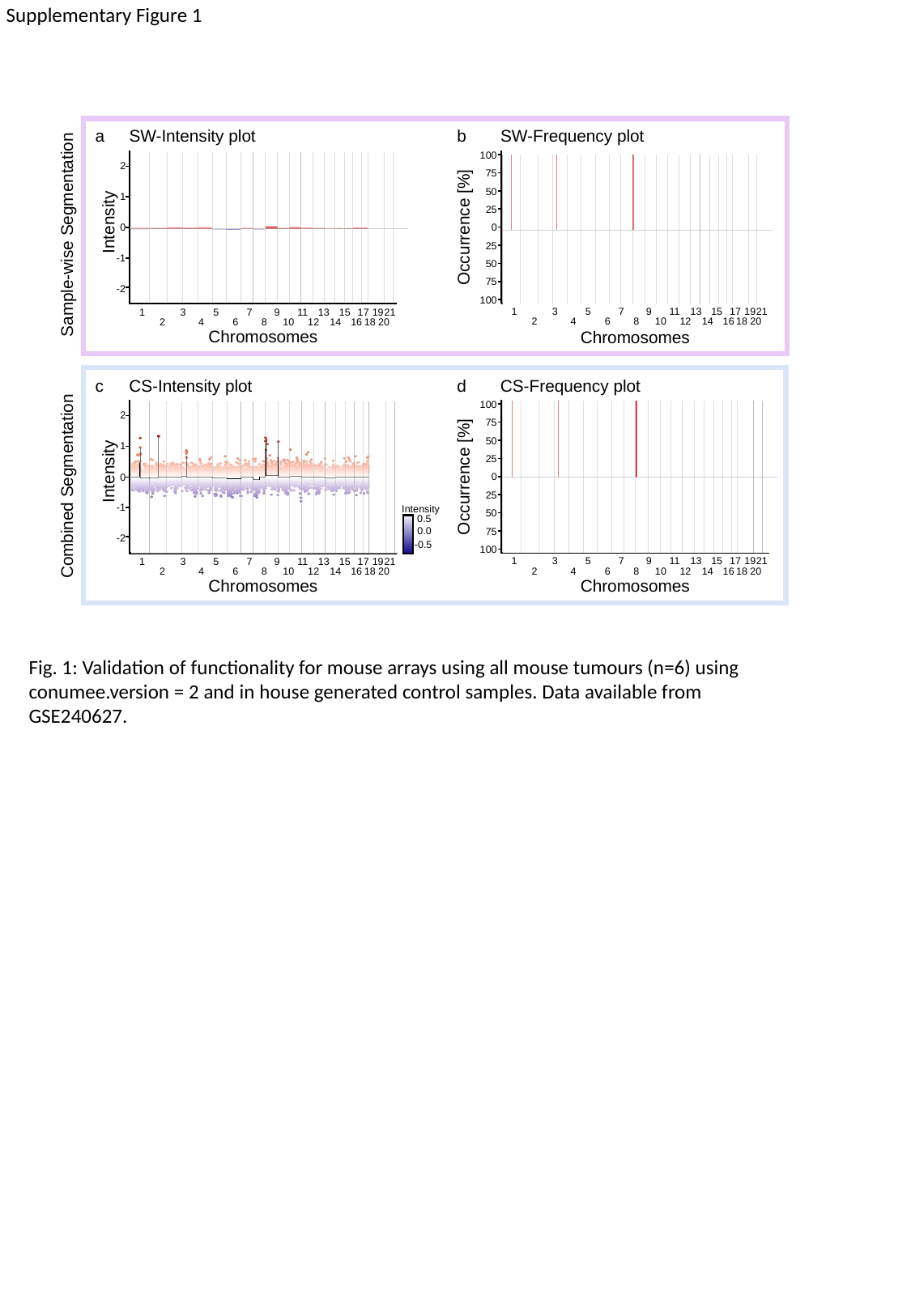

Supplementary Figure 1
a
SW-Intensity plot
b
SW-Frequency plot
100
75
50
25
0
25
50
75
100
Occurrence [%]
1
3
5
7
9
11
13
15
17
19
21
2
4
6
8
10
12
14
16
18
20
Chromosomes
2
1
Intensity
0
Sample-wise Segmentation
-1
-2
1
3
5
7
9
11
13
15
17
19
21
2
4
6
8
10
12
14
16
18
20
Chromosomes
c
CS-Intensity plot
d
CS-Frequency plot
100
75
50
25
0
25
50
75
100
Occurrence [%]
1
3
5
7
9
11
13
15
17
19
21
2
4
6
8
10
12
14
16
18
20
Chromosomes
2
1
Intensity
0
Combined Segmentation
-1
Intensity
0.5
 0.0
-0.5
-2
1
3
5
7
9
11
13
15
17
19
21
2
4
6
8
10
12
14
16
18
20
Chromosomes
Fig. 1: Validation of functionality for mouse arrays using all mouse tumours (n=6) using conumee.version = 2 and in house generated control samples. Data available from GSE240627.

## Slide 2
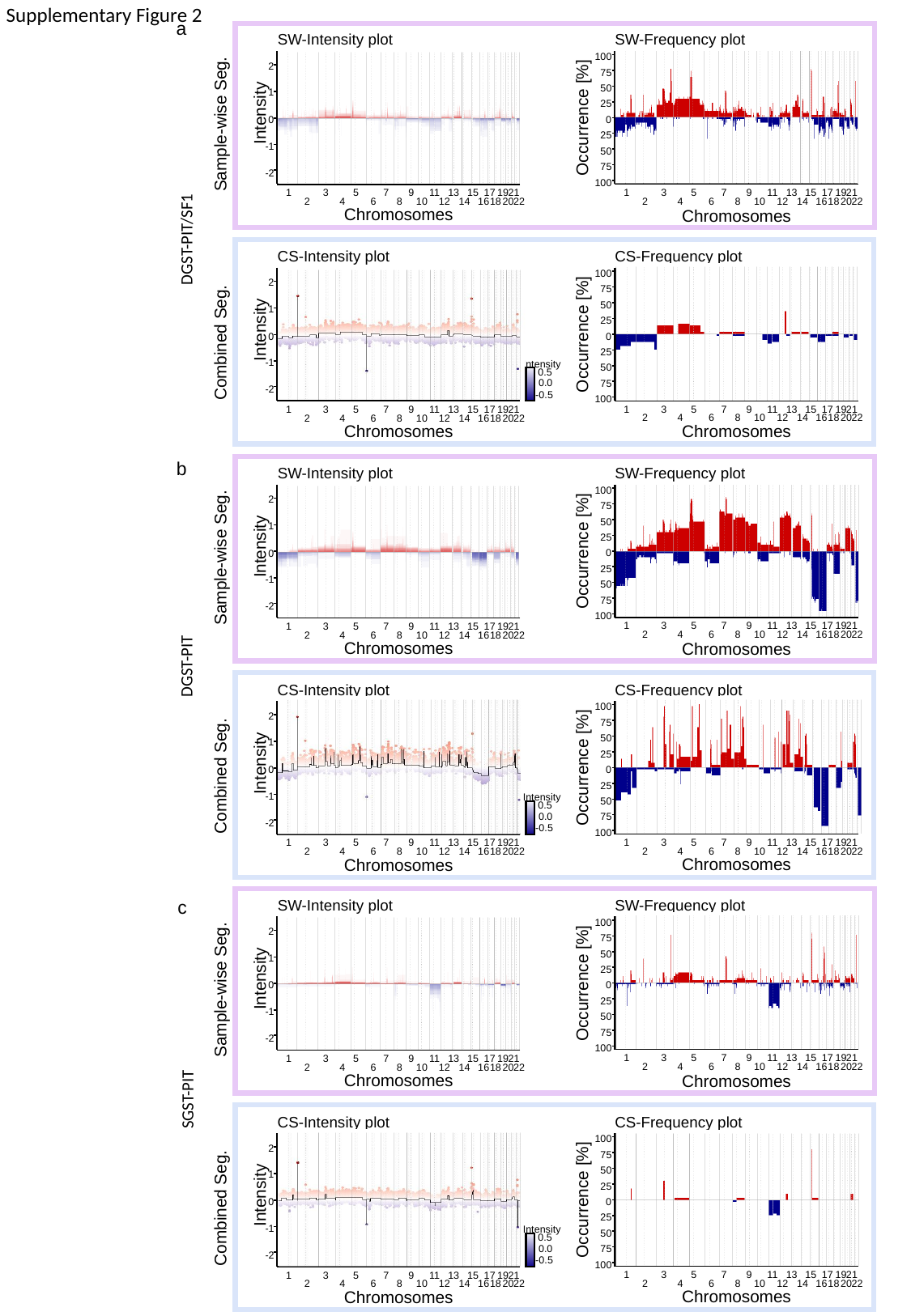

Supplementary Figure 2
a
SW-Intensity plot
SW-Frequency plot
100
75
50
25
0
25
50
75
100
Occurrence [%]
1
3
5
7
9
11
13
15
17
19
21
2
4
6
8
10
12
14
16
18
20
22
Chromosomes
2
1
Intensity
0
Sample-wise Seg.
-1
-2
1
3
5
7
9
11
13
15
17
19
21
2
4
6
8
10
12
14
16
18
20
22
Chromosomes
CS-Intensity plot
CS-Frequency plot
100
75
50
25
0
25
50
75
100
Occurrence [%]
1
3
5
7
9
11
13
15
17
19
21
2
4
6
8
10
12
14
16
18
20
22
Chromosomes
2
1
Intensity
0
Combined Seg.
-1
Intensity
0.5
 0.0
-0.5
-2
1
3
5
7
9
11
13
15
17
19
21
2
4
6
8
10
12
14
16
18
20
22
Chromosomes
DGST-PIT/SF1
b
SW-Intensity plot
SW-Frequency plot
100
75
50
25
0
25
50
75
100
Occurrence [%]
1
3
5
7
9
11
13
15
17
19
21
2
4
6
8
10
12
14
16
18
20
22
Chromosomes
2
1
Intensity
0
Sample-wise Seg.
-1
-2
1
3
5
7
9
11
13
15
17
19
21
2
4
6
8
10
12
14
16
18
20
22
Chromosomes
CS-Intensity plot
CS-Frequency plot
100
75
50
25
0
25
50
75
100
Occurrence [%]
1
3
5
7
9
11
13
15
17
19
21
2
4
6
8
10
12
14
16
18
20
22
Chromosomes
2
1
Intensity
0
Combined Seg.
-1
Intensity
0.5
 0.0
-0.5
-2
1
3
5
7
9
11
13
15
17
19
21
2
4
6
8
10
12
14
16
18
20
22
Chromosomes
DGST-PIT
c
SW-Intensity plot
SW-Frequency plot
100
75
50
25
0
25
50
75
100
Occurrence [%]
1
3
5
7
9
11
13
15
17
19
21
2
4
6
8
10
12
14
16
18
20
22
Chromosomes
2
1
Intensity
0
Sample-wise Seg.
-1
-2
1
3
5
7
9
11
13
15
17
19
21
2
4
6
8
10
12
14
16
18
20
22
Chromosomes
CS-Intensity plot
CS-Frequency plot
100
75
50
25
0
25
50
75
100
Occurrence [%]
1
3
5
7
9
11
13
15
17
19
21
2
4
6
8
10
12
14
16
18
20
22
Chromosomes
2
1
Intensity
0
Combined Seg.
-1
Intensity
0.5
 0.0
-0.5
-2
1
3
5
7
9
11
13
15
17
19
21
2
4
6
8
10
12
14
16
18
20
22
Chromosomes
SGST-PIT

## Slide 3
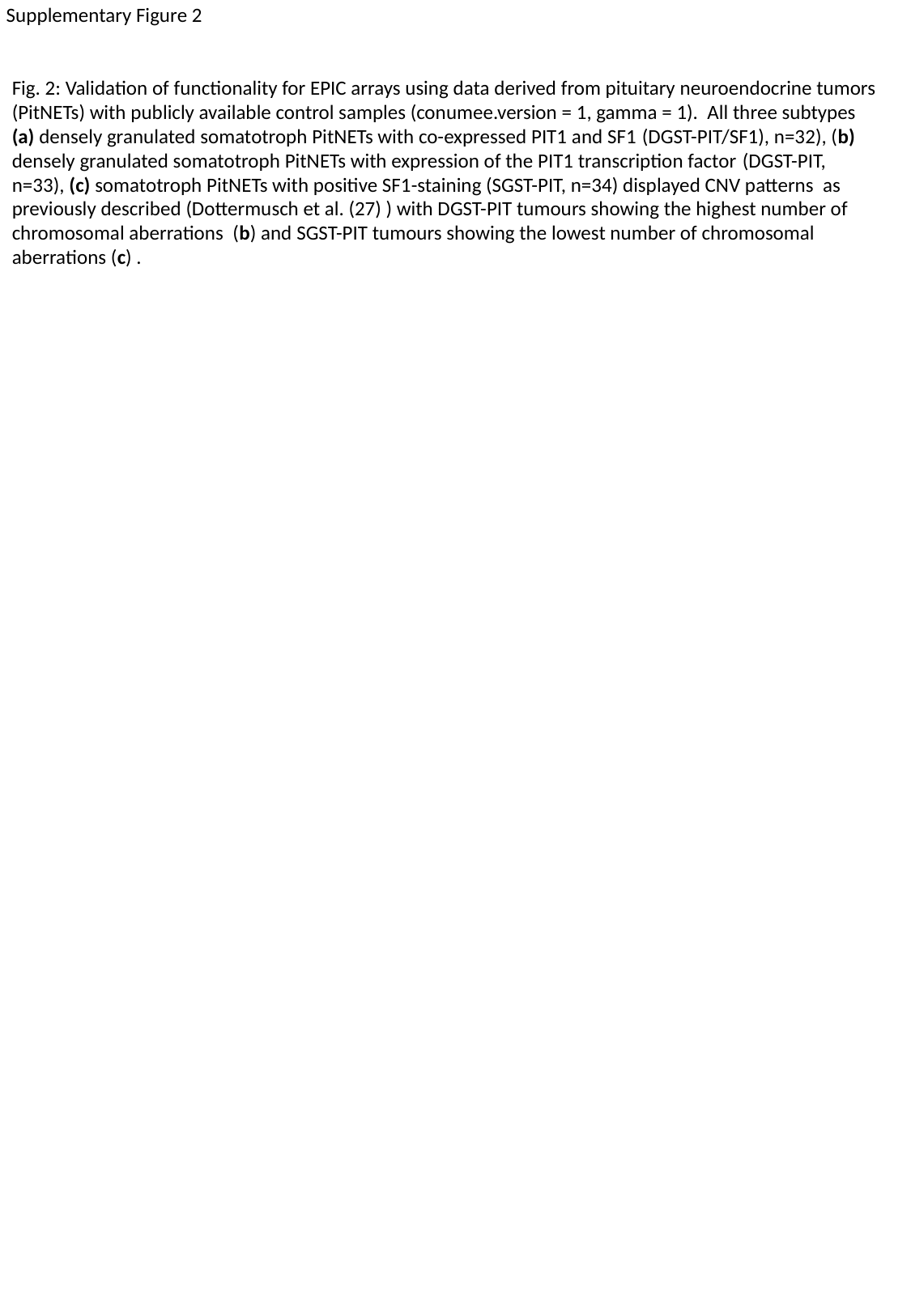

Supplementary Figure 2
Fig. 2: Validation of functionality for EPIC arrays using data derived from pituitary neuroendocrine tumors (PitNETs) with publicly available control samples (conumee.version = 1, gamma = 1). All three subtypes (a) densely granulated somatotroph PitNETs with co-expressed PIT1 and SF1 (DGST-PIT/SF1), n=32), (b) densely granulated somatotroph PitNETs with expression of the PIT1 transcription factor (DGST-PIT, n=33), (c) somatotroph PitNETs with positive SF1-staining (SGST-PIT, n=34) displayed CNV patterns as previously described (Dottermusch et al. (27) ) with DGST-PIT tumours showing the highest number of chromosomal aberrations (b) and SGST-PIT tumours showing the lowest number of chromosomal aberrations (c) .

## Slide 4
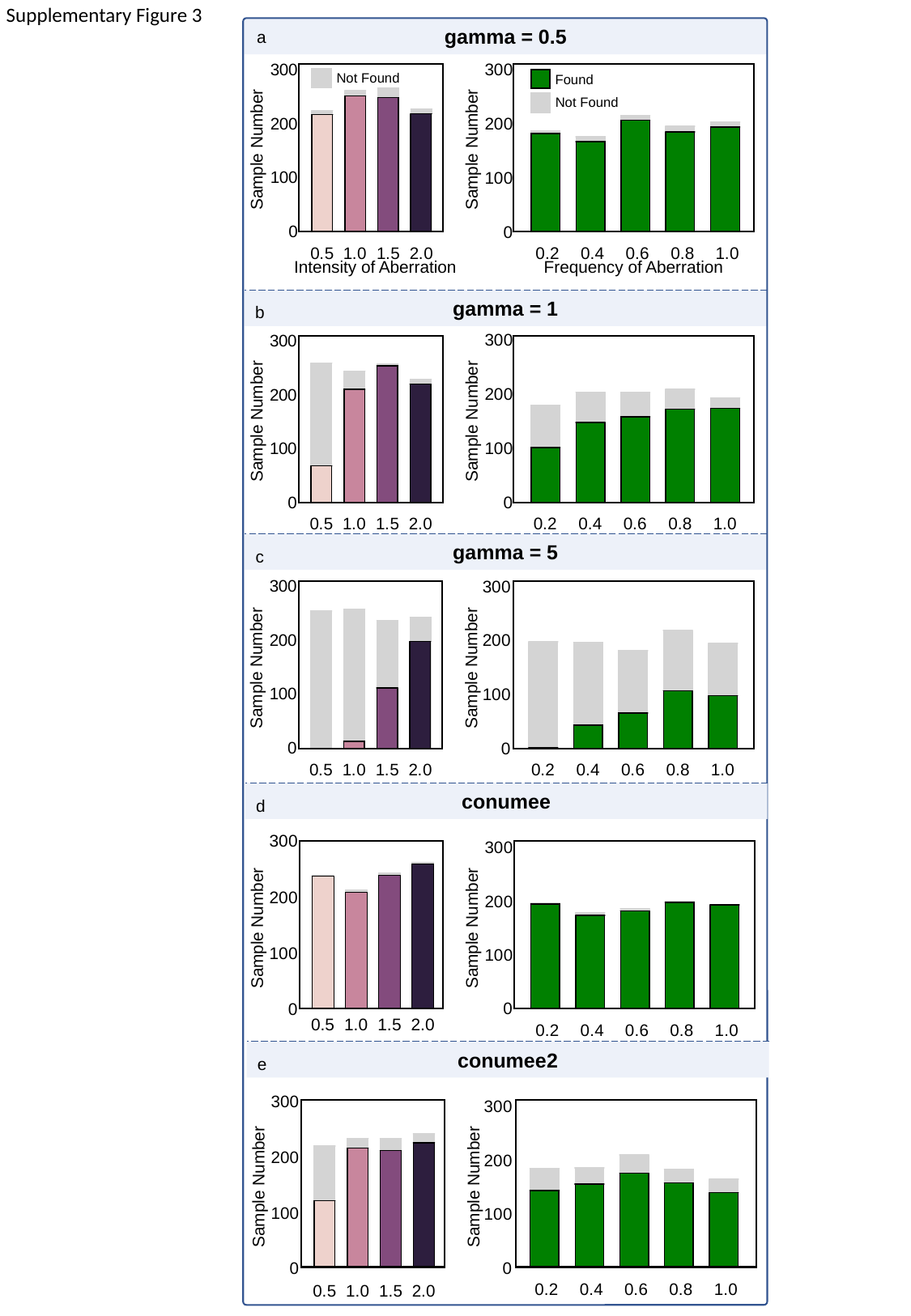

Supplementary Figure 3
gamma = 0.5
a
Not Found
Found
Not Found
Sample Number
Sample Number
Intensity of Aberration
Frequency of Aberration
gamma = 1
b
Sample Number
Sample Number
gamma = 5
c
Sample Number
Sample Number
conumee
d
Sample Number
Sample Number
conumee2
e
Sample Number
Sample Number

## Slide 5
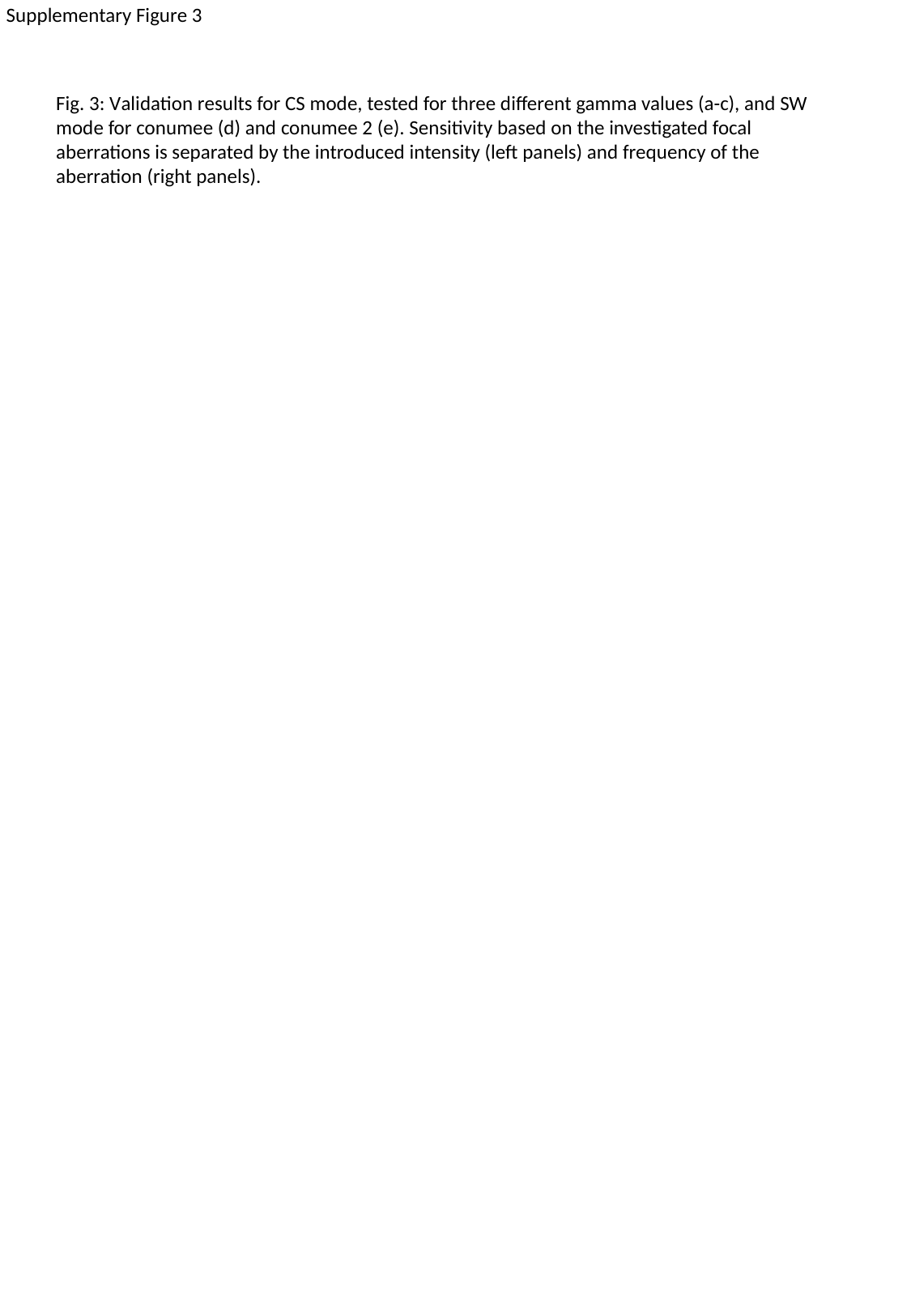

Supplementary Figure 3
Fig. 3: Validation results for CS mode, tested for three different gamma values (a-c), and SW mode for conumee (d) and conumee 2 (e). Sensitivity based on the investigated focal aberrations is separated by the introduced intensity (left panels) and frequency of the aberration (right panels).

## Slide 6
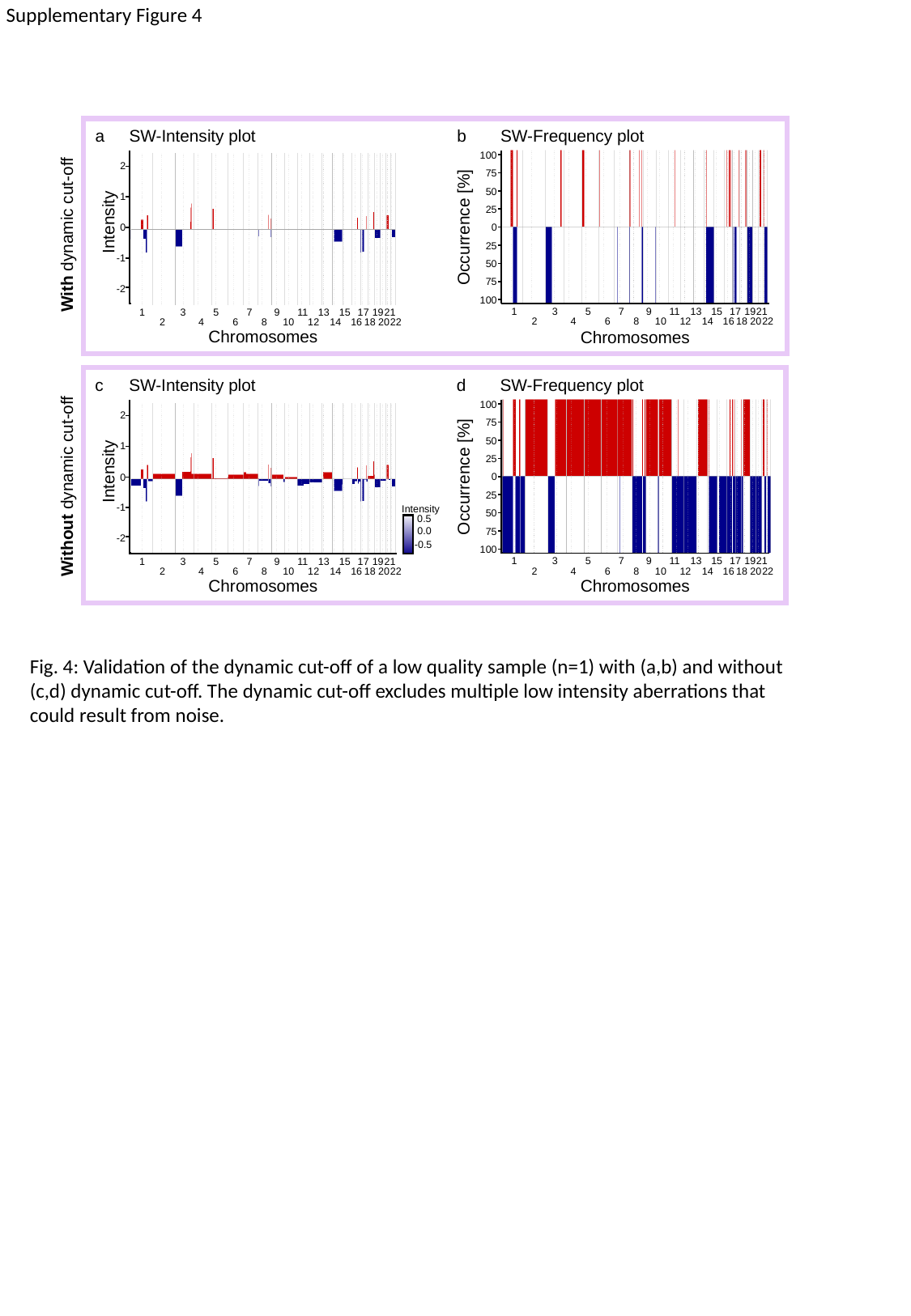

Supplementary Figure 4
a
SW-Intensity plot
b
SW-Frequency plot
100
75
50
25
0
25
50
75
100
Occurrence [%]
1
3
5
7
9
11
13
15
17
19
21
2
4
6
8
10
12
14
16
18
20
22
Chromosomes
2
1
Intensity
0
With dynamic cut-off
-1
-2
1
3
5
7
9
11
13
15
17
19
21
2
4
6
8
10
12
14
16
18
20
22
Chromosomes
c
SW-Intensity plot
d
SW-Frequency plot
100
75
50
25
0
25
50
75
100
Occurrence [%]
1
3
5
7
9
11
13
15
17
19
21
2
4
6
8
10
12
14
16
18
20
22
Chromosomes
2
1
Intensity
0
Without dynamic cut-off
-1
Intensity
0.5
 0.0
-0.5
-2
1
3
5
7
9
11
13
15
17
19
21
2
4
6
8
10
12
14
16
18
20
22
Chromosomes
Fig. 4: Validation of the dynamic cut-off of a low quality sample (n=1) with (a,b) and without (c,d) dynamic cut-off. The dynamic cut-off excludes multiple low intensity aberrations that could result from noise.
